# Development of PSMA-Targeted Liposomal Zinc for Prostate Cancer Therapy

**DOI:** 10.3390/nano16120705

**Published:** 2026-06-08

**Authors:** Sujan Kumar Mondal, Elizabeth Kenyon, Alexander L. Klibanov, Anna Moore

**Affiliations:** 1 Precision Health Program, Michigan State University, 766 Service Road, East Lansing, MI 48824, USA; mondalsu@msu.edu (S.K.M.); kenyonel@msu.edu (E.K.); 2 Department of Radiology, College of Human Medicine, Michigan State University, 846 Service Road, East Lansing, MI 48824, USA; 3 Department of Medicine, University of Virginia, Davis Wing, Hospital Drive, Suite 5357, Charlottesville, VA 22904, USA; sasha@virginia.edu

**Keywords:** prostate cancer, zinc, liposomal delivery, prostate-specific membrane antigen (PSMA)

## Abstract

Normal prostate epithelial cells accumulate high intracellular zinc levels that maintain optimum mitochondrial metabolism and proliferation. Prostate cancer cells lose this zinc-accumulating capacity, enabling metabolic reprogramming that supports tumor progression. Restoring intracellular zinc selectively in prostate tumors represents a promising therapeutic strategy; however, systemic zinc administration is limited by the inability of prostate cancer cells to take up free zinc resulting from ZIP1 transporter downregulation. To overcome this challenge, we developed a formulation of prostate-specific membrane antigen (PSMA)-targeted, zinc-loaded liposomes (Zn-TL) to enable tumor-selective intracellular zinc delivery. Zn-TL was prepared with uniform nanoscale size, low polydispersity, and negative surface charge. The formulation showed minimal zinc leakage during storage and sustained retention in vitro. In prostate cancer cells, Zn-TL demonstrated receptor-mediated uptake, resulting in increased cytotoxicity and apoptosis. In vivo, we performed proof-of-principle studies showing prolonged circulation and tumor accumulation of Zn-TL in mice bearing PSMA-positive tumors. While tumor growth was delayed during early and intermediate stages of tumor development, this effect diminished at later stages. The stage-dependent efficacy suggests that Zn-TL may be most effective when used earlier in disease progression. These results also suggest that Zn-TL represents a promising platform for metabolic intervention and may benefit from combination strategies to enhance efficacy in advanced disease.

## 1. Introduction

Prostate cancer (PC) is the second-leading cancer diagnosis among men after skin cancer in the United States and remains a major contributor to cancer-related morbidity and healthcare burden [1,2,3]. More than 300,000 new cases were estimated in 2025, accounting for nearly 35,000 reported deaths [1]. Despite improvements in early PC detection and treatment, the clinical management of prostate cancer, particularly advanced and metastatic disease, continues to be challenging [4]. For localized tumors, treatment options often include active surveillance, radical prostatectomy, or radiation therapy [5]. In contrast, patients with advanced or recurrent disease typically require androgen-deprivation therapy followed by chemotherapy or radiotherapy [5,6], both of which are associated with substantial systemic toxicity [3,7,8] and recurrence of castration-resistant PC [4,9]. These challenges highlight an urgent need for innovative therapeutic approaches that are mechanistically distinct from androgen-targeted strategies, capable of overcoming resistance mechanisms, and safer than current systemic treatments.

Normal prostate epithelial cells accumulate high levels of intracellular zinc, largely mediated by zinc transporter (ZIP1) [10]. These high levels of zinc inhibit mitochondrial aconitase (m-aconitase), the enzyme responsible for the conversion of citrate to isocitrate, the first step for citrate oxidation in the Krebs cycle [11]. As a result, normal prostate epithelial cells rely on a truncated Krebs cycle, resulting in an approximately 65% reduction in ATP yield from glucose oxidation. The normal prostate epithelial cells compensate this ATP loss by a high aerobic glycolysis [12]. However, downregulation of ZIP1 in prostate cancer substantially limits the zinc accumulation in malignant prostate cells [11,13,14]. This leads to a dramatic 70–90% reduction in intracellular zinc levels, even at early stages of malignancy [15]. The zinc concentration in the malignant peripheral prostate, which is the main region of cancer development, is 0.4 vs. 3 mM in normal prostate epithelial cells [16]. With zinc no longer available to inhibit m-aconitase, citrate is efficiently oxidized through a fully functional Krebs cycle, resulting in 38 ATP/glucose compared to 14 ATP/glucose resulting from the aerobic oxidation of glucose in normal prostate cells [11]. Thus, cancer cells become energy-efficient, in contrast to the energy-inefficient prostate epithelial cells, and change their phenotype to a “citrate-oxidizing” phenotype that supports tumor growth and survival [11]. Importantly, the constitutive level of m-aconitase is essentially the same in normal and cancer cells, and the difference in citrate production is due to the decreased zinc levels [17].

This unique biology suggests that restoration of zinc levels in prostate cancer cells would lead to inhibition of m-aconitase, disruption of the existing metabolic advantage, and induction of energy starvation, leading to cell death. Previous attempts to therapeutically deliver zinc to prostate cancer using oral zinc supplementation [11] or systemic administration of zinc salt [18,19] have been largely unsuccessful due to inefficient internalization of zinc resulting from ZIP1 transporter downregulation. Direct intratumoral injection of zinc acetate [20] also failed to produce convincing antitumor effects due to zinc transporter downregulation. Continuous zinc sulfate infusion via subcutaneously implanted osmotic pumps [21] lacks clinical relevance, in addition to the inability of prostate cancer cells to take up free zinc.

To overcome these limitations, an alternative strategy is needed, one that bypasses ZIP1-mediated uptake and delivers zinc directly into cancer cells. The prostate-specific membrane antigen (PSMA) provides an attractive targeting mechanism. PSMA is a transmembrane glycoprotein that is markedly upregulated in prostate cancer, both in primary and metastatic sites, while maintaining minimal expression in normal tissues [22,23,24,25]. Its selective expression pattern and high internalization efficiency have enabled its successful use in imaging [26,27], targeted drug delivery [28], and radioligand therapies [29]. Leveraging PSMA for targeted zinc delivery could maximize intracellular zinc accumulation in tumor cells while minimizing off-target effects.

In this study, we developed a PSMA-targeted liposomal zinc formulation (Zn-TL) with encapsulated zinc, capable of delivering it directly into PSMA-expressing prostate cancer cells. This approach bypasses the limitations of zinc toxicity and ZIP1 downregulation, while allowing effective tumor accumulation of zinc following systemic administration. By delivering zinc, we aim to truncate the Krebs cycle, suppress ATP production, and induce metabolic arrest resulting in prostate cancer cell death. An additional advantage of our targeted liposomal platform is its capacity for image-guided delivery using optical imaging, enabling real-time, non-invasive monitoring of biodistribution and tumor uptake of the formulation. Using in vitro uptake assays, metabolic activity measurements, and in vivo pharmacokinetic and efficacy studies in PC3-PIP tumor-bearing mice, we demonstrate that Zn-TL liposomes deliver zinc to PSMA-expressing prostate cancer cells and reduce tumor viability. Our findings provide proof of concept for a novel metabolic intervention strategy for prostate cancer and establish the foundation for further development of PSMA-targeted zinc therapies.

## 2. Materials and Methods

### 2.1. Synthesis and Characterization of DSPE-PEG3400-PSMA-Peptide

DSPE-PEG3400-PSMA-peptide was synthesized by reacting the primary amino group in PSMA targeting peptide, N-[[[(1S)-5-amino-1-carboxypentyl]amino]carbonyl]-L-glutamic acid (hereafter referred to as PSMA-peptide) with the NHS group in DSPE-PEG3400-NHS following previously reported methods with slight modification [30,31,32]. An aliquot of 58.5 mg PSMA-peptide (180 μmol, A2B Chemicals, San Diego, CA, USA, #BC58874) and 100 mg DSPE-PEG3400-NHS (30 μmol, NOF America Corporation, White Plains, NY, USA #DSPE-034GS) were dissolved in 10 mL dry DMSO, followed by the addition of 70 µL of DIPEA to this mixture. The reaction was continued at room temperature for 48 h with stirring, and the mixture was then transferred to a dialysis bag (3.5 KDa MWCO, Repligen, Waltham, MA, USA #132720T). The reaction mixture was dialyzed sequentially against PBS buffer (2 times, 1000 mL, 4 h each) and deionized water (3 times, 1000 mL, 4 h each). The dialyzed sample was then lyophilized at –5 °C to obtain the final product, DSPE-PEG3400-PSMA-peptide. The control lipid, DSPE-PEG3400-ASP3, was prepared following a method similar to that described above by conjugating tri-aspartic acid (H-Asp-Asp-Asp-OH, herein referred to as ASP3, Fisher Scientific, Hampton, NH, USA #50-383-436) with DSPE-PEG3400-NHS. The synthesized product was identified using MALDI-TOF MS and ^1^H-NMR. The product was stored at –20 °C until use.

### 2.2. Liposome Preparation

Liposomes were prepared using the reverse-phase evaporation method. Briefly, phospholipids [1,2-distearoyl-sn-glycero-3-phosphocholine (DSPC) or 1,2-dioleoyl-sn-glycero-3-phosphocholine (DOPC)], cholesterol, DSPE-mPEG(2000), and either DSPE-PEG(3400)-PSMA-peptide or DSPE-PEG(3400)-ASP3 were dissolved at varying molar ratios in a 4 mL mixture of chloroform and diethyl ether (1:2, *v*/*v*). For in vitro and in vivo studies, DiD and DiR fluorescent dyes were incorporated into the lipid mixture at a molar ratio of 0.01 relative to DSPC/DOPC.

Subsequently, 1 mL of 300 mM zinc sulfate solution (pH 5.5, unbuffered) was added to the lipid mixture, followed by sonication for 1 min using a Branson 450 Sonifier (100% duty cycle) to obtain a water-in-oil emulsion. The organic solvents were immediately removed using a rotary evaporator, resulting in the formation of liposomes. The liposomes were further size-reduced and homogenized by sequential extrusion through polycarbonate membranes with 0.4 µm, 0.2 µm, and 0.1 µm pore sizes using a mini-extruder (Avanti Polar Lipids, Alabaster, AL, USA), performing 10 passes for each membrane. Unencapsulated Zn^2+^ ions were removed by passing the liposomes through a PD-10 desalting column (Cytiva, Marlborough, MA, USA) using saline as the eluent. The liposome suspension was subsequently concentrated using a centrifugal concentrator with a 10 KDa molecular weight cut-off membrane.

For quantification of zinc encapsulation, the liposomes were digested with concentrated nitric acid, and the zinc content was determined using inductively coupled plasma optical emission spectrometry (ICP-OES). Zinc encapsulation efficiency and loading capacity were calculated using the following equations:

Encapsulation efficiency (EE%) = (amount of Zn^+2^ encapsulated in the liposomes/amount of Zn^+2^ used) × 100%.

Drug loading content (DLC%) = [amount of Zn^+2^ encapsulated in the liposomes/(Amount of Zn^+2^ encapsulated in the liposomes + amount of the total lipids)] × 100%.

Drug loading efficiency (DLE%) = (actual Zn^+2^ loading content/theoretical Zn^+2^ loading content) × 100%.

### 2.3. Zeta Potential and Size Measurement

The hydrodynamic diameter, polydispersity index (PDI), and zeta potential of the zinc-loaded liposomes were determined by the dynamic light scattering (DLS) method using a Zetasizer Ultra instrument (Malvern, UK). All measurements were performed in triplicate, and the data are presented as the mean ± SD.

### 2.4. Transmission Electron Microscopy (TEM)

The morphological characterization of liposomes was performed using transmission electron microscopy (JEOL 1400, Tokyo, Japan). Briefly, liposome samples (1 mM) were applied onto carbon-coated copper grids and allowed to air-dry. The grids were subsequently stained with a 2% (*w*/*v*) aqueous solution of uranyl acetate to enhance contrast. Imaging was carried out using a transmission electron microscope (JEOL 1400, Japan).

### 2.5. In Vitro Zinc Release and Leakage Study

The zinc release kinetics from the liposomes were evaluated using a dialysis-based diffusion method. Briefly, 200 μL of zinc-loaded liposome suspension was applied into dialysis inserts with a molecular weight cut-off (MWCO) of 3.5 KDa (Slide-A-Lyzer MINI, Thermo Scientific, Hampton, NH, USA). Each dialysis insert was placed into a glass vial containing 20 mL of either phosphate-buffered saline (PBS, pH 7.4) or acetate buffer (10 mM, pH 5.2) to simulate physiological and acidic conditions, respectively. The vials were incubated at 37 °C with gentle agitation. At predetermined time intervals, 500 μL of the external buffer (dialysate) was collected and replaced with an equal volume of fresh buffer. The zinc concentration in the dialysate was quantified using ICP-OES. The cumulative percentage of zinc released was calculated and expressed as a function of time.

To assess zinc leakage from liposomes under storage conditions, zinc-loaded liposomes were incubated at 4 °C. At predetermined time points, 10 μL of the liposome suspension was withdrawn and diluted to 100 μL with acetate buffer (10 mM, pH 5.2). The diluted liposome suspension was then centrifuged using a 100 KDa MWCO (Amicon Ultra, MilliporeSigma, Burlington, MA, USA) at 5000× *g* for 10 min. The filtrate was collected, and the zinc content was determined by ICP-OES.

### 2.6. Cell Culture

Human prostate cancer cell lines PC3-PIP (PSMA^+^) and PC3-Flu (PSMA^–^) were obtained from Dr. Martin G. Pomper of University of Texas Southwestern Medical Center [31,33,34]. Cells were cultured in RPMI1640 medium containing 10% fetal bovine serum and 1% penicillin streptomycin supplemented with 2 μg/mL puromycin.

### 2.7. Fluorescence Microscopy of Cellular Uptake

For the uptake study, 3 × 10^3^ PC3-PIP cells in 100 μL complete RPMI medium were seeded on glass cover slips in a 96-well plate. After 48 h of incubation, medium was replaced with Dil dye-labeled liposomes in RPMI medium, which were then incubated for 2 h. Next, cells were washed twice with PBS and fixed by incubating with 4% paraformaldehyde (PFA) for 10 min at 37 °C. After that, PFA was removed, and cells were washed with PBS three times. Glass cover slips with cells were mounted on the slide using a DAPI-containing mounting medium (Southern Biotech, Birmingham, AL, USA) and imaged under a Nikon Eclipse microscope (Nikon Instruments Inc., Melville, NY, USA) at 40× magnification.

### 2.8. Quantification of Intracellular Zinc Accumulation

To quantify the intracellular zinc accumulation PC3-PIP cells were seeded in a 12-well plate (1.5 × 10^5^ cells/well) and treated with increasing concentrations of Zn-TL or Zn-CL formulations or saline (control). After 2 h of treatment, the medium was removed, the cells were harvested using trypsin and the cell number/well was counted. Cells were collected by centrifugation and digested with concentrated nitric acid to extract intracellular zinc. The zinc content was quantified using ICP-OES, and the results were expressed as picograms (pg) of zinc per cell.

### 2.9. Intracellular Zinc Staining

For intracellular zinc staining, 4000 cells were seeded into poly-L-lysine-coated 16-well chamber slides (Nunc™ Lab-Tek™ Chamber Slide System, Thermo Fisher Scientific, Hampton, NH, USA, Cat. No. 178599PK) and incubated with saline or DiD-labeled Zn-TL or Zn-CL liposomes at 72 μM. After 2 h of incubation, the treatment medium was replaced with fresh complete growth medium, followed by an additional 4 h incubation period. Upon completion of the treatment, intracellular zinc staining was performed using 6 μM FluoZin™-3, AM (Invitrogen, Carlsbad, CA, USA, Cat. No. F24195) prepared in 0.02% Pluronic F-127. Cells were incubated with the staining solution at 37 °C for 30 min. Subsequently, cells were washed and incubated with phenol red-free complete RPMI medium for 30 min at 37 °C to allow de-esterification of the dye. Live-cell images were acquired using an EVOS fluorescence microscope (Thermo Fisher Scientific) at 20× magnification.

### 2.10. Flow Cytometry

Quantitative cellular uptake was performed using flow cytometry. Briefly, PC3-PIP or PC3-Flu cells were seeded in a 12-well plate (1.5 × 10^5^ cells/well) and treated with DiD-labeled Zn-TL or Zn-CL liposomes at a final DiD dye concentration of 100 ng/mL. For PSMA-specific blocking studies, cells were pre-incubated with free PSMA peptide (at 0, 1, 10, 100 μM final concentration) for 2 h prior to liposome addition. After 2 h of incubation with the liposomes, cells were harvested using trypsin and washed with PBS. Fluorescence intensity was measured using a Cytek Aurora spectral flow cytometer (Cytek Biosciences, Fremont, CA, USA), and data were analyzed using FlowJo software (v10, BD Biosciences, San Jose, CA, USA).

### 2.11. Protein Corona Studies

To evaluate the effect of protein corona formation on liposome targeting, DiD-labeled Zn-TL liposomes (200 ng DiD equivalent) were incubated with either saline or 55% FBS or mouse serum at 37 °C for 1 h with constant agitation to allow protein corona formation. The 55% serum concentration was selected to match physiological serum concentration. For cellular uptake studies, PC3-PIP cells were seeded in a 12-well plate (1.5 × 10^5^ cells/well) in 1 mL RPMI medium. After 12 h, cells were pre-incubated with free PSMA peptide (100 μM final concentration) for 2 h. Next, corona-preformed liposomes were added directly to cells at a final DiD concentration of 100 ng/mL. After 2 h of incubation, cells were harvested using trypsinization, washed with PBS, and fixed with 4% paraformaldehyde. Fluorescence intensity was measured using a Cytek Aurora spectral flow cytometer (Cytek Biosciences), and data were analyzed using FlowJo software (BD Biosciences).

### 2.12. Cytotoxicity Assay

The cytotoxic effect of zinc-loaded liposomes was assessed using an MTS assay (Promega, Madison, WI, USA). Briefly, PC3-PIP or PC3-Flu cells were seeded in a 96-well plate (2 × 10^3^ cells/well) in 100 µL of RPMI medium. The following day, cells were either left untreated or incubated with increasing concentrations of Zn-CL or Zn-TL formulations (80, 160, 320, or 640 µM). After 72 h of incubation, the treatment medium was removed, and cells were incubated with 20% MTS reagent prepared in RPMI medium for 1 h. Absorbance was measured at 490 nm using a microplate reader (SpectraMax iD5, Molecular Devices, San Jose, CA, USA). Wells without cells were included as blanks, and absorbance values were normalized to the corresponding blanks. Cell viability was expressed relative to the untreated control.

### 2.13. Apoptosis Study

The apoptotic effect of zinc-loaded liposomes (Zn-TL and Zn-CL) was evaluated using an Annexin V apoptosis assay (Invitrogen, Cat. No. 88-8102-72) in PC3-PIP cells. PC3-PIP cells were seeded at a density of 1.5 × 10^5^ cells per well in 6-well plates. Next day, cells were treated with Zn-CL or Zn-TL at an equivalent zinc concentration of 160 µM, whereas control cells were treated with saline for 36 h. Next, cells were harvested, washed with cold PBS, and stained with Annexin V–PE and 7-aminoactinomycin D (7-AAD) according to the manufacturer’s instructions. Stained cells were then analyzed using a Cytek Aurora spectral flow cytometer. Data analysis was performed using FlowJo software (v10, BD Biosciences, San Jose, USA). Apoptotic cells were defined as the combined population of Annexin V^+^/7-AAD^−^ (early apoptosis) and Annexin V^+^/7-AAD^+^ (late apoptosis), while dead cells were defined as Annexin V^−^/7-AAD^+^ [35].

### 2.14. Cellular ATP Quantification

Intracellular ATP levels were quantified using the CellTiter-Glo^®^ Luminescent Cell Viability Assay (Promega) according to the manufacturer’s instructions. Briefly, PC3-PIP cells were seeded in a 96-well plate (2 × 10^3^/well). Next day, cells were treated with Zn-TL, Zn-CL, or saline control. Following 24 h incubation, an equal volume of CellTiter-Glo reagent was added, and luminescence (relative light units, RLU) was measured using a plate reader. ATP levels were expressed as percent relative luminescence units (RLU) normalized to saline-treated controls.

### 2.15. Biodistribution Study

For biodistribution analysis, contralateral subcutaneous flank tumors were established in male nude mice (6-week-old nu/nu; The Jackson Laboratory, Bar Harbor, ME, USA) by injecting cells suspended in 100 μL of a 1:1 mixture of DPBS and Matrigel. Three million PC3-PIP (PSMA^+^) cells were injected in the left flank, and 1 million PC3-Flu (PSMA^−^) cells were injected in the right flank. A three-fold higher number of PC3-PIP cells was implanted to account for their slower proliferation rate relative to PC3-Flu as described in [36]. Tumor dimensions were measured using digital calipers, and tumor volumes (mm^3^) were calculated using the formula: V = 0.52 × L × W × H. Once tumors reached approximately 100 mm^3^, mice were intravenously administered DiR-labeled Zn-TL (n = 5) or Zn-CL (n = 4) liposomes at a dose of 5.6 mg/kg zinc. Twenty-four hours later, mice were euthanized, and organs and tumors were excised. Zinc accumulation in tissues was quantified by ICP-OES as described before and expressed as the percentage of the injected dose per gram of tissue (%ID/g). To further assess liposomal accumulation in tumors, cryopreserved tumor specimens were sectioned into 10 μm sections, fixed with 4% paraformaldehyde, and mounted using DAPI Fluoromount-G (SouthernBiotech, Birmingham, AL, USA). Tumor sections were imaged under a ZEISS Axioscan 7 scanner (Carl Zeiss Microscopy GmbH, Jena, Germany) at 20× magnification in DAPI and Cy7 channels. Image acquisition and analysis were performed with Zeiss Zen software (v3, Carl Zeiss Microscopy GmbH, Jena, Germany).

At the completion of the therapeutic study (described below), organ distribution of DiR-labeled Zn-TL was evaluated as well in a similar fashion. Excised organs were imaged using a whole-body in vivo fluorescence imaging (FLI) system (IVIS Spectrum whole-body scanner, Revvity, Waltham, MA, USA). The percent of recovered dose per gram tissue (%RD/g) was calculated after multiple injections by adding the radiance value for all recovered organs and calculating the percentage for each organ.

### 2.16. Pharmacokinetic (PK) Analysis

Pharmacokinetic studies were conducted in PC3-PIP tumor-bearing mice. Briefly, 3 × 10^6^ PC3-PIP cells suspended in Matrigel as described above were subcutaneously implanted into the right flank of 6-week-old male mice (nu/nu; Jackson Laboratory, n = 5). After 2 weeks of tumor growth, mice received an intravenous injection of Zn-TL liposomes at a zinc-equivalent dose of 5.0 mg/kg. Blood samples were collected at predetermined time points (0, 0.08, 0.75, 6, 12, 24, and 72 h post-injection) via tail vein nicking using a 25G needle (BD Biosciences, San Jose, CA, USA). Samples were transferred to anticoagulant-coated tubes (Microvette CB300, Sarstedt Inc., Newton, NC, USA) and centrifuged at 2000× *g* for 10 min at 4 °C to separate plasma. Plasma samples were digested with concentrated nitric acid and analyzed for zinc concentration using ICP-OES. Plasma zinc concentrations were normalized to baseline (pre-injection) levels, and pharmacokinetic parameters were determined using PKSolver software (v2.0, China Pharmaceutical University, Nanjing, China) [37].

### 2.17. In Vivo Therapeutic Study

The therapeutic potential of zinc-loaded liposomes was evaluated in mice bearing PC3-PIP tumors implanted in the right flank, as described above. Tumor growth was monitored twice weekly using digital calipers, and tumor volume was calculated accordingly. When tumor volumes reached approximately 100 mm^3^ (Day 13), mice were randomized into three groups: Zn-TL liposome injected group (n = 11; 5 mg/kg zinc), control zinc sulfate in saline injected group (n = 5; 1 mg/kg zinc) and control saline solution injected group (n = 5). Due to toxicity, we were unable to administer ZnSO_4_ at a 5 mg zinc/kg dose; mice injected with this dose died immediately post-injection. Treatments were administered intravenously twice weekly and continued until tumors reached a volume of 2000 mm^3^.

Survival outcomes were assessed using Kaplan–Meier analysis with two surrogate endpoints: (a) time to reach a five-fold increase in tumor volume from the treatment starting point and (b) time to reach a tumor volume of 2000 mm^3^. Statistical significance was determined by log-rank (Mantel–Cox) test. Upon reaching the second endpoint, mice were sacrificed, and major organs and tumors were collected for ex vivo fluorescence imaging and subsequent analyses. For zinc quantification, tumor samples were digested in concentrated nitric acid (HNO_3_) and analyzed by ICP-OES.

All animals were housed in the standard MSU Animal Facility under supervision of full-time animal caretakers. The MSU Animal Facility is under supervision of the MSU Office of Laboratory Animal Research, directed by Diane Ferguson, DVM. All animal experiments including pain management were conducted in accordance with the Declaration of Helsinki, and the protocol (PROTO20200322) was approved by the Institutional Animal Care and Use Committee (IACUC) at Michigan State University on 18 January 2021.

### 2.18. Statistical Analysis

All results are presented as mean ± SD unless specified otherwise. Statistical analysis was performed using GraphPad Prism 10 software. *p* Values of less than 0.05 were considered statistically significant.

## 3. Results

### 3.1. Synthesis of the Lipid–Peptide Conjugates

**Figure 1:**
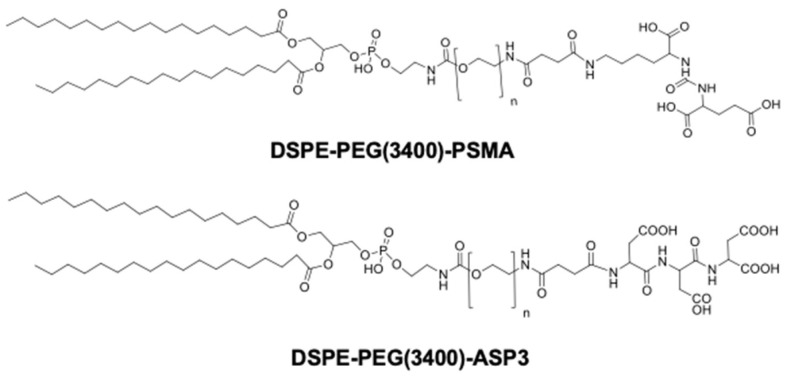
Chemical structure of PSMA ligand peptide conjugate with DSPE-PEG(3400) lipid, DSPE-PEG(3400)-PSMA, and tri-aspartic acid conjugate with DSPE-PEG(3400) lipid, DSPE-PEG3400-ASP3.

To prepare PSMA targeting zinc-loaded liposome, first, DSPE-PEG3400-PSMA (Figure 1) was synthesized by reacting the primary amino group in PSMA peptide ligand (Figure S1A) with the NHS group of DSPE-PEG3400-NHS. The unreacted molecules were removed by dialysis followed by lyophilization to obtain a white solid final product, DSPE-PEG3400-PSMA. Control lipid, DSPE-PEG3400-ASP3 (Figure 1), was prepared following a similar method by conjugating tri-aspartic acid (ASP3, Figure S1A) with DSPE-PEG3400-NHS. The synthesized product was characterized using ^1^H-NMR and MALDI-TOF MS (Figure S1).

Compared with DSPE-PEG-NHS, the DSPE-PEG-PSMA conjugate showed a clear reduction in the characteristic NHS proton signal, along with the appearance of new peaks corresponding to the PSMA peptide, including urea proton resonances, confirming successful amide bond formation (Figure S1B). Similarly, the DSPE-PEG-ASP3 spectrum displayed diminished NHS proton intensity and the appearance of ASP3-specific signals, further supporting successful conjugation (Figure S1C). MALDI-TOF mass spectrometry provided additional confirmation of lipid–peptide conjugation (Figure S1D). Both DSPE-PEG-PSMA and DSPE-PEG-ASP3 showed an increase in molecular mass compared to DSPE-PEG-NHS lipid, consistent with peptide attachment. Collectively, these analyses verified the successful synthesis of both targeting and control lipid–peptide conjugates. The purified products were stored at −20 °C until further use.

### 3.2. Preparation of Zinc-Loaded Liposome and Its Characterizations

**Figure 2:**
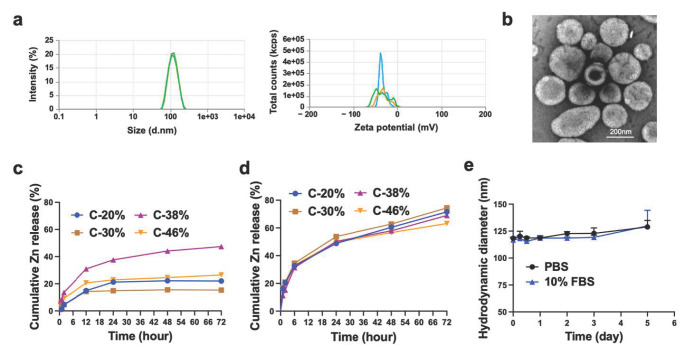
(**a**) Representative hydrodynamic size and zeta potential of Zn-TL. (**b**) Transmission electron microscopy image of Zn-TL liposomes. Scale bar = 200 nm. Cumulative zinc release in acetate buffer pH 5.5 from zinc-loaded liposomes, where main lipids were either (**c**) DSPC or (**d**) DOPC with varying amounts of cholesterol (20, 30, 38 or 46 mol%) while keeping other lipid amounts the same. (**e**) Stability evaluation of Zn-TL liposomes by analyzing hydrodynamic size measurement in PBS and 10% FBS over 5 days.

The synthesized conjugate was used to prepare the zinc-loaded liposome by the reverse-phase evaporation method. To evaluate the impact of liposome membrane rigidity on zinc-release properties, liposomes were formulated using either DSPC or DOPC. DSPC, a fully saturated lipid with a high phase transition temperature, forms rigid and tightly packed bilayers, whereas DOPC contains unsaturated chains that form more fluid membranes. The liposomes prepared with DSPC were named as Zn-TL_DS or Zn-CL_DS, whereas DOPC lipid-based liposomes were named as Zn-TL or Zn-CL, where TL and CL represented targeting liposome-carrying DSPE-PEG3400-PSMA and control liposome-carrying DSPE-PEG3400-APS3, respectively. The resultant liposome had a mean hydrodynamic diameter in the range of 120–140 nm, PDI 0.1 to 0.2, and the zeta potential in PBS was around –35 mV (Table 1 and Figure 2). Transmission electron microscopy imaging showed the liposomal structure (Figure 2b) of Zn-TL. In Zn-TL liposome, zinc encapsulation efficiency and loading efficiency were 3.8% and 5.6%, respectively (Table 1). Next, Zn-release analysis in acetate buffer (pH 5.5) showed that Zn-TL_DS liposome had a low amount of Zn^2+^ release, with 18–40% of total Zn^2+^ released in 72 h in all the tested Zn-TL_DS (varying cholesterol content, Figure 2c) compared to the Zn-TL liposome, which had more than 60% of total Zn^2+^ release in 72 h in all the tested Zn-TL liposome (Figure 2d). Based on these results, we selected DOPC lipid-based liposomes for all future studies. Size (Figure 2e) and PDI (Figure S2A) of the Zn-TL liposomes in PBS and 10% FBS buffer did not change significantly over 5 days, confirming the stability of this liposome formulation in these buffers. Further Zn^2+^ release from DOPC-based liposomes was evaluated under different physiologically relevant conditions (Figure S2B). A higher release was noticed at pH 5.5, with ~70% cumulative release over 72 h (~30% within 6 h), whereas minimal release (~5%) was observed in PBS (pH 7.4) or 10% FBS-containing buffer over 72 h. These results indicate that Zn^2+^ release from the liposomes is enhanced under acidic conditions, while remaining relatively stable at neutral pH. Although in vitro release conditions may not fully mimic the in vivo environments, the observed pH-dependent trend is consistent with the potential for enhanced Zn^2+^ release in acidic intracellular compartments such as endosomes. To test Zn^2+^ leakage from the liposome, we estimated the free Zn^2+^ amount in Zn-TL at various time points and found that ~5% and ~10% of total zinc leaked out after 2 and 21 days, respectively, at 4 °C (Figure S2C). In aggregate, we successfully prepared a zinc-loaded liposome formulation that was stable with low PDI and with a size of around 120 nm.

### 3.3. PSMA Specific Uptake and Intracellular Localization of Liposomes

**Figure 3:**
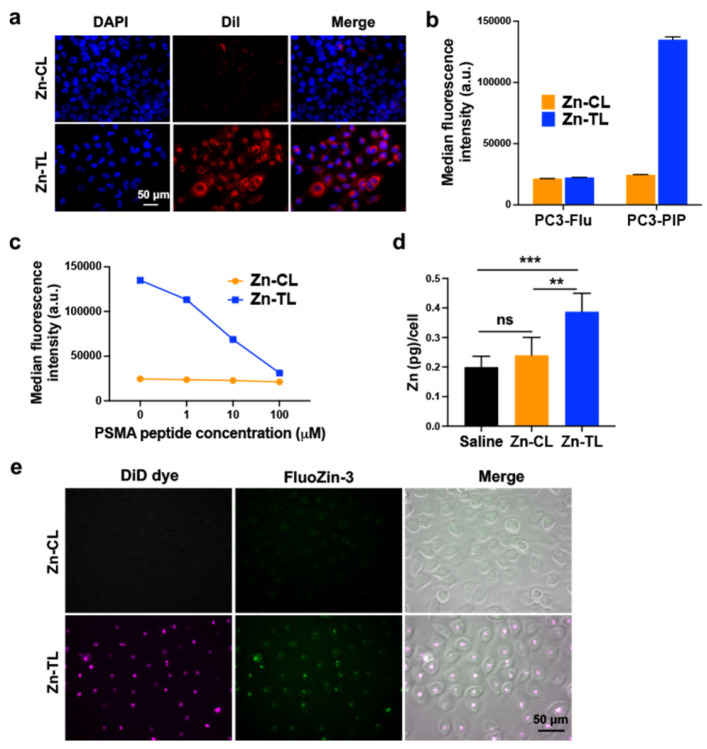
(**a**) Microscopic images of PC3-PIP cells representing uptake of Zn-TL and Zn-CL liposomes. Dil = Red, DAPI stained nuclei = blue; scale bar = 50 μm. (**b**) Flow cytometric analysis of Zn-TL or Zn-CL liposomes in PC3-FLU and PC3-PIP cells (n = 3). (**c**) PSMA selectivity study: PC3-PIP or PC3-FLU cells were pretreated with different amounts of PSMA-specific peptide for 2 h, followed by coincubation with Zn-TL or Zn-CL liposomes. Uptake was assessed by flow cytometry (n = 3). (**d**) Zinc accumulation in PC3-PIP cells following coincubation with ZnSO_4_, Zn-TL, and Zn-CL (n = 5). (**e**) Microscopic analysis of zinc accumulation in PC3-PIP cells following incubation with Zn-CL or Zn-TL liposomes. Zinc accumulated in cells was stained with FluoZin-3, followed by microscopy in GFP channel. Scale bar = 50 μm. The data presented in (**b**–**d**) are mean ± SD. ns = non-significant, ** *p* < 0.01, *** *p* < 0.001.

Following liposome formulation preparation, we first optimized the molar ratio of DSPE-PEG-PSMA required for maximal cellular uptake. Fluorescence microscopy showed a progressive increase in intracellular Dil-labeled Zn-TL liposomes in PC3-PIP cells with increasing DSPE-PEG-PSMA fraction in the liposome formulation (Figure S3A). A similar trend was further confirmed by flow cytometry-based quantitative cellular uptake assay (Figure S3B). The highest uptake of Zn-TL was observed at 2 mol% of DSPE-PEG-PSMA, and subsequent experiments were performed using this formulation. Representative fluorescence microscopy images and flow cytometry analysis (Figure 3a,b) demonstrate substantial uptake of Zn-TL liposomes by PSMA-positive PC3-PIP cells compared to Zn-CL liposomes. In contrast, PSMA-negative PC3-Flu cells showed comparably negligible uptake of both liposomal formulations (Figure 3b). Further, a competitive assay in PC3-PIP cells with increasing concentrations of free PSMA peptide reduced the uptake of Zn-TL liposomes (Figure 3c), completely blocking it at 100 µM free PSMA peptide and confirming that the uptake was PSMA-mediated. Zinc accumulation estimated by ICP-OES was significantly higher in PC3-PIP cells for Zn-TL liposomes (0.39 pg/cell) compared to untreated controls (0.20 pg/cell) or cells treated with Zn-CL liposomes (0.24 pg/cell) (Figure 3d). Consistently, intracellular zinc staining revealed pronounced fluorescence in Zn-TL-treated PC3-PIP cells compared to cells treated with Zn-CL (Figure 3e), demonstrating enhanced zinc delivery by targeted liposomes. Furthermore, co-localization of DiD dye with the FluoZin-3 staining confirmed the stability of the formulation during the incubation.

It is well known that when liposomes encounter biological fluids, proteins and other biomolecules form a “protein corona” around their surface, which can alter their function and interfere with interactions between targeting ligands and tissues, potentially reducing the efficacy of targeted delivery systems [38,39,40]. To assess the functional impact of protein corona formation on cellular uptake and PSMA-ligand engagement, we pre-incubated Zn-TL with 55% FBS or mouse serum to allow a protein corona to form. We then investigated Zn-TL cellular uptake and the availability of the PSMA ligand on the cell surface. The results of our studies demonstrated that Zn-TL liposomes with pre-formed corona showed slightly reduced cellular uptake compared to Zn-TL liposomes (Figure S4). Nevertheless, pre-treatment of cells with free PSMA peptide reduced the uptake of corona-pre-formed Zn-TL liposomes to background levels, confirming that the PSMA ligand remained available for engagement and functional on the liposome surface despite protein corona formation. These results confirmed that, although serum proteins likely adsorb onto the liposome surface, PSMA receptor-mediated targeting remains largely preserved.

### 3.4. PSMA-Targeting Zn^2+^-Loaded Liposomes Selectively Kill PSMA-Expressing Cells

**Figure 4:**
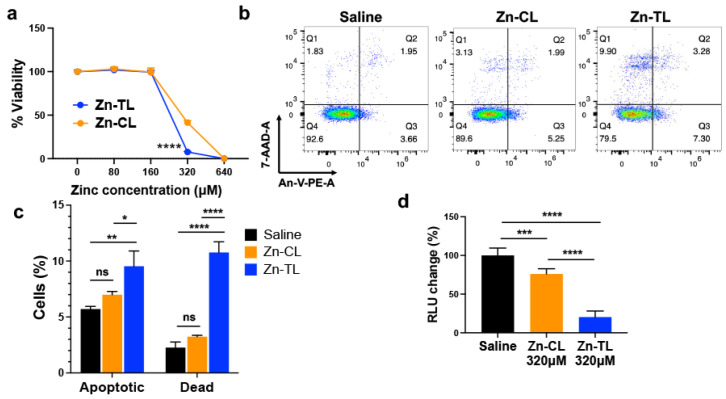
Effect of Zn-TL and Zn-CL liposomes on prostate cancer cells. (**a**) Cell viability of PC3-PIP cells was assessed by MTS assay after 72 h of incubation with Zn-TL or Zn-CL liposomes (n = 4–5). (**b**) Representative Annexin V/7-AAD flow cytometry showing apoptosis in PC3-PIP cells following 36 h treatment with saline, Zn-CL, or Zn-TL. (**c**) Quantitative analysis of apoptotic cells (early and late apoptosis combined) and dead cells following treatment (n = 3). (**d**) Intracellular ATP levels, expressed as percent change in relative luminescence units (RLU) relative to control, were measured in PC3-PIP cells following 24 h treatment with Zn-TL or Zn-CL (n = 6). Data are presented as mean ± SD for panels (**a**–**d**). Statistical analysis was performed using two-way ANOVA followed by Tukey’s multiple comparison test for panel (**a**) and one-way ANOVA followed by Tukey’s multiple-comparison test for panels (**c**,**d**). **** *p* < 0.0001; *** *p* < 0.001; ** *p* < 0.01; * *p* < 0.05; ns, not significant.

To explore the cytotoxic effect of Zn^2+^-loaded liposomes, PC3-PIP cells were incubated with Zn-TL and Zn-CL liposomes for 72 h. The viability assay demonstrated that both Zn-TL and Zn-CL liposome treatments reduced cell viability (Figure 4a). However, Zn-TL treatment reduced cell viability to 7% compared to treatment with Zn-CL resulting in 42% viability. Analysis of apoptosis (Figure 4b) revealed significantly higher levels of Annexin V staining in Zn-TL-treated cells compared to Zn-CL-treated cells (9.5% vs 7%; Figure 4c). In addition, Zn-TL treatment resulted in a substantially greater proportion of dead cells (10.7%) relative to Zn-CL (3.2%) (Figure 4c). These results indicate that PSMA-targeted zinc delivery promotes enhanced cancer cell death through both apoptotic and non-apoptotic mechanisms.

To further evaluate the metabolic impact of zinc delivery, intracellular ATP levels were quantified in PC3-PIP cells. Treatment with Zn-CL resulted in a modest 24% reduction in ATP levels, whereas cells treated with Zn-TL produced a markedly greater 80% reduction in cellular ATP compared to control saline-treated cells (Figure 4d). These findings indicate that PSMA-targeted zinc delivery induced substantial energy depletion, consistent with zinc-mediated mitochondrial dysfunction. The increased ATP reduction observed with Zn-TL supports enhanced intracellular zinc delivery through receptor-mediated uptake.

### 3.5. Biodistribution and Pharmacokinetic Profile of PSMA-Targeting Liposomes in Tumor-Bearing Mice

**Figure 5:**
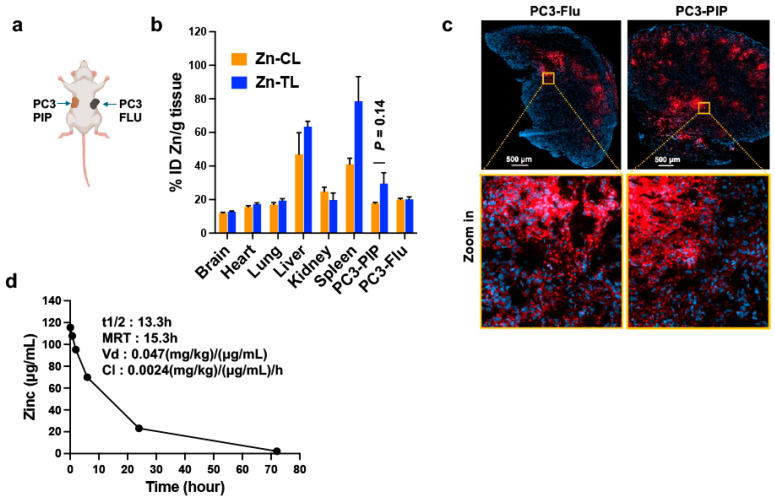
(**a**) Schematic illustrating the subcutaneous PC3-PIP and PC3-FLU flank tumors. (**b**) Zinc amount was estimated by ICP-OES 24 h after Zn-TL administration and is presented as percent of injected dose per gram tissue (%ID/g). Data are presented as mean ± SEM (n = 5 for Zn-TL; n = 4 for Zn-CL). Mann–Whitney test was used for statistical analysis. (**c**) Fluorescence microscopy of tumor tissues showing the presence of DiR-labeled liposomes in the tumor tissue. Blue—DAPI nuclear stain nuclei; red—DiR-labeled liposomes. Scale bar = 500 μm. (**d**) Pharmacokinetic analysis showing the presence of zinc (μg/mL) in plasma at different time points following i.v. administration of Zn-TL liposomes (n = 5).

**Figure 6:**
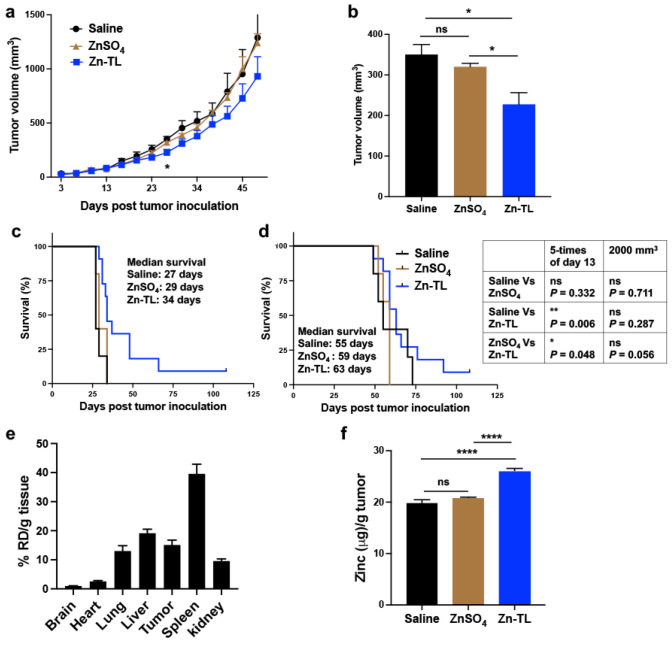
In vivo therapeutic efficacy of zinc-loaded PSMA-targeted liposomes (Zn-TL) in tumor-bearing mice. (**a**) Tumor growth curves of mice treated with saline (n = 5), free zinc (ZnSO_4_, 1 mg Zn/kg; n = 5), or Zn-loaded PSMA-targeted liposomes (Zn-TL, 5 mg Zn/kg; n = 11). Treatments began when tumors reached ~100 mm^3^ (Day 13). Statistical analysis was performed using two-way ANOVA followed by Tukey’s multiple comparisons test. (**b**) Bar graph representing tumor volumes at Day 27, highlighting group differences at this representative time point. (**c**,**d**) Kaplan–Meier survival analysis of surrogate endpoints (saline n = 5, ZnSO_4_ n = 5, Zn-TL n = 11): (**c**) Time to five-fold increase from treatment start time and (**d**) time to reach a tumor volume of 2000 mm^3^. The corresponding statistical analysis is summarized in the adjacent table. (**e**) Percentage of recovered dose per gram tissue (%RD/g) at the study endpoint (tumor volume ~2000 mm^3^). The recovered dose was calculated by summing the total radiant efficiency of the major organs. (**f**) Quantification of zinc accumulation in tumors at endpoint (2000 mm^3^), measured by ICP-OES and expressed as micrograms zinc per gram of tumor tissue (n = 10). All data are presented as mean ± SEM. One-way ANOVA followed by Tukey’s multiple comparisons test was used for statistical analysis. ns = not significant; * *p* < 0.05; ** *p* < 0.01; **** *p* < 0.0001.

After confirming Zn-TL PSMA specificity and efficacy at the cellular level, we evaluated the ability of Zn-TL and Zn-CL liposomes to deliver zinc in a mouse prostate cancer model. Mice with contralateral PC3-PIP and PC3-Flu tumors (Figure 5a) were intravenously injected with Zn-TL or Zn-CL liposomes. After 24 h, mice were sacrificed, and zinc accumulation in vital organs and tumors was evaluated by the ICP analysis of zinc content. While both formulations demonstrated comparable accumulation in PSMA-negative PC3-Flu tumors, we observed an increased trend of zinc accumulation in PSMA-positive PC3-PIP tumors, though it did not reach statistical significance (Figure 5b). This is likely due to the small tumor volume (~100 mm^3^) at the time of the injection, where limited vascular permeability may have restricted nanoparticle extravasation and thus reduced the impact of receptor-mediated uptake [41,42]. Notably, in a therapeutic study described below, we observed a substantial tumoral accumulation of Zn-TL liposomes, comparable to liver levels, in a larger-volume tumor (~2000 mm^3^) (Figure 6e and Figure S5), suggesting that enhanced vascular permeability in advanced tumors facilitated improved nanoparticle delivery and target-specific retention. As expected, we observed significant accumulation of the liposomal formulations in the organs of the reticuloendothelial system (RES), with the highest accumulation in the liver, consistent with the data from other investigators [43,44]. Fluorescence microscopy of excised tumors confirmed accumulation of the liposomes after intravenous injection (Figure 5c). Overall, this biodistribution study indicates that an appreciable amount of zinc-liposome is reaching the tumor tissue and localizing with tumor cells.

Following our observation of enhanced accumulation of Zn-TL liposomes in PC3-PIP tumor compared to Zn-CL liposomes, we chose this formulation for the in vivo studies evaluating its pharmacokinetics and therapeutic efficacy. We first assessed the in vivo stability of the encapsulated zinc payload. Although minimal zinc leakage was detected under the in vitro conditions, we reasoned that the small molecular size of Zn^2+^ could lead to zinc loss during systemic circulation. To rule out significant zinc leakage from the liposomes in vivo, we conducted a pharmacokinetic (PK) study in mice bearing PSMA-positive PC3-PIP prostate tumors. Zn^2+^-loaded PSMA-targeted Zn-TL liposomes were administered intravenously, and plasma zinc levels were monitored over 72 h post-injection. The Zn-TL formulation demonstrated favorable pharmacokinetic behavior characterized by prolonged circulation, as evidenced by an elimination half-life of 13.3 h and a mean residence time (MRT) of 15.3 h. The relatively low volume of distribution (Vd) (0.047 (mg/kg)/(μg/mL)) suggests that the formulation remains largely within the vascular compartment, consistent with a long-circulating carrier. In addition, the low systemic clearance (Cl) (0.0024 (mg/kg)/(μg/mL)/h) indicates slow elimination, supporting sustained systemic exposure and the potential for enhanced tumor accumulation (Figure 5d). These results indicate prolonged circulation of the Zn^2+^-loaded liposomes, suggesting that zinc remained substantially encapsulated over the duration of the study. The low clearance and small volume of distribution are consistent with nanoparticle-bound Zn^2+^, further supporting the integrity of the liposomal formulation during systemic exposure.

### 3.6. Anti-Tumor Effect of PSMA-Targeting Zn-TL in a Prostate Tumor Mouse Model

Next, we evaluated the therapeutic efficacy of zinc-loaded PSMA-targeted liposomes (Zn-TL) in mice bearing PSMA-expressing PC3-PIP tumors. When tumor volumes reached approximately 100 mm^3^ (Day 13), mice were intravenously injected twice weekly with Zn-loaded PSMA-targeted liposomes, free zinc (as ZnSO_4_), or saline solution. The study continued until tumors reached a volume of 2000 mm^3^. The results of this study demonstrated slower growth in Zn-TL-treated mice compared to those in the saline or ZnSO_4_ groups (Figure 6a). Zn-TL was specifically effective at retarding tumor growth during the early phase of therapy, as indicated by a significantly smaller tumor volume on Day 27 compared to the other groups (Figure 6b). At a later time point, Zn-TL–treated mice exhibited smaller tumors; however, the differences were not statistically significant.

To assess the therapeutic impact of Zn-TL across different stages of tumor progression, we performed Kaplan–Meier survival analyses based on surrogate endpoints: (1) time to reach a five-fold increase in initial tumor volume (from Day 13; Figure 6c) and (2) time to reach a tumor volume of 2000 mm^3^ (Figure 6d). Zn-TL treatment significantly delayed the time to five-fold tumor growth compared to both saline and ZnSO_4_-treated groups (*p* = 0.006 and *p* = 0.048, respectively). The median times to reach this endpoint were 27 days for saline, 29 days for ZnSO_4_, and 34 days for Zn-TL groups. Although not statistically significant, the median times to reach 2000 mm^3^ were 55 days for saline, 59 days for ZnSO_4_, and 63 days for the Zn-TL group, pointing to the increased efficacy with the Zn-TL treatment.

Ex vivo fluorescence imaging (Figure 6e and Figure S5) at the study endpoint (tumor volume ~2000 mm^3^) showed substantial accumulation of Zn-TL liposomes in PSMA-expressing tumors, at levels comparable to that in liver tissue. Consistent with this, zinc quantification by ICP-OES revealed significantly higher zinc accumulation in tumors from Zn-TL–treated mice compared to both saline and ZnSO_4_ groups (Figure 6f), supporting the enhanced tumor-specific delivery of zinc via Zn-TL.

In summary, Zn-loaded PSMA-targeted liposomes demonstrated efficient accumulation in PSMA-expressing prostate tumors and significantly inhibited tumor growth during the early phase of therapy. However, therapeutic efficacy at later stages was limited, highlighting the potential benefit of combining Zn-TL with existing prostate cancer treatments to improve outcomes in all stages of disease progression.

## 4. Discussion

Zinc plays a well-established yet paradoxical role in prostate cancer biology. While normal prostate epithelium accumulates high intracellular zinc levels that suppress mitochondrial metabolism and proliferation, malignant prostate cells lose this zinc-accumulating capacity, due to downregulation of zinc-transporter (ZIP1) conferring a metabolic advantage that supports tumor progression [12,45,46,47]. Restoring intracellular zinc selectively in prostate cancer cells therefore represents an attractive therapeutic strategy; however, systemic zinc administration is ineffective due to poor tumor specificity and dose-limiting toxicity. In this study, we addressed these challenges by developing a PSMA-targeted, zinc-loaded liposomal delivery platform (Zn-TL) designed to bypass the biological bottleneck associated with ZIP1 loss, and by systematically evaluating its physicochemical properties, tumor-targeting capability, pharmacokinetics, and therapeutic efficacy. While various delivery systems are being developed [48,49], our choice for using a liposomal delivery platform was based on its favorable pharmacokinetics, reduced systemic toxicity, good solubility, and formulation flexibility. The biocompatibility and biodegradability of liposomes generally provide improved safety profiles and reduced immunogenicity compared to many systemic carriers.

We successfully synthesized DSPE-PEG3400-PSMA lipid–peptide conjugates and incorporated them into stable zinc-loaded liposomes with favorable physicochemical characteristics, including a uniform size (~120–140 nm), low polydispersity, and negative surface charge (Table 1 and Figure 2). These properties are well suited for prolonged circulation and tumor accumulation via the enhanced permeability and retention (EPR) effect [50,51,52,53], while PEGylation [54] and cholesterol [55] incorporation further contributed to formulation stability. Importantly, Zn-TL liposomes showed minimal zinc leakage during long-term storage and sustained zinc retention in vitro (Figure S2B,C), addressing a major concern associated with delivering small, highly diffusible metal ions. Interestingly, DOPC-based liposomes showed substantially higher Zn release in acidic medium over DSPC-based liposomes. Although DSPC, being fully saturated, provides greater structural rigidity to the liposome membrane, it also limits zinc release even under acidic conditions mimicking the endosomal compartment (pH~5–5.5), as evidenced by the 18–40% release observed for Zn-TL_DS formulations (Figure 2c). In contrast, DOPC, containing one unsaturated acyl chain, maintains a relatively fluid bilayer that allows more membrane permeability, resulting in >60–70% cumulative release at pH 5.5 within 72 h (Figure 2d). Importantly, this release was not accompanied by premature zinc leakage at physiological pH in either PBS- or FBS-containing buffer (Figure S2B), nor was leakage observed during storage (Figure S2C), indicating systemic zinc retention prior to cellular uptake. Together, these properties support that the DOPC-based formulation provides the optimal balance between cargo retention during circulation and efficient zinc release in acidic intracellular compartments, and therefore, DOPC-based liposomes were elected for all subsequent study.

At the cellular level, Zn-TL exhibited robust PSMA-specific uptake in PC3-PIP cells compared to the uptake in PSMA-negative PC3-Flu (Figure 3), confirming receptor-mediated internalization. Functionally, PSMA-targeted zinc delivery translated into enhanced cytotoxicity in PSMA-expressing cells (Figure 4a). Zn-TL induced significantly greater loss of cell viability in PC3-PIP cells compared to control Zn-CL liposomes, at equivalent zinc concentrations. We further showed that Zn-TL promoted higher levels of both apoptotic and non-apoptotic cell death (Figure 4b,c), suggesting that targeted zinc accumulation amplifies intracellular stress beyond apoptotic signaling alone. These findings support the concept that receptor-mediated zinc delivery selectively induces tumor-suppressive effects in PSMA-expressing prostate cancer cells.

To explore the metabolic consequences of targeted zinc delivery, intracellular ATP levels were assessed following treatment. The pronounced reduction in ATP observed in cells treated with Zn-TL suggests that receptor-mediated zinc accumulation induces acute energetic stress. Given the established role of zinc in disrupting mitochondrial metabolism particularly through inhibition of enzymes involved in the tricarboxylic acid cycle, this energy depletion is likely a consequence of zinc-mediated impairment of mitochondrial function. Such metabolic disruption would be expected to reduce ATP production, promote bioenergetic insufficiency, and sensitize cells to apoptosis and other forms of cell death. While the precise mitochondrial targets were not directly interrogated in this study, the marked ATP depletion observed with Zn-TL supports the concept that targeted zinc delivery perturbs tumor cell metabolism.

Biodistribution studies of the formulations showed accumulation of both targeted and non-targeted liposomes in tumors, which is in accordance with the EPR effect. However, while biodistribution studies conducted in small (~100 mm^3^) tumors showed only a trend toward enhanced zinc accumulation in PSMA-positive tumors that did not reach statistically significant difference (Figure 5b), later therapeutic studies revealed substantial tumor accumulation of Zn-TL in larger (~2000 mm^3^) tumors, reaching levels comparable to that in liver tissue (Figure 6e). This observation is consistent with increased vascular permeability and nanoparticle extravasation in more advanced tumors, allowing receptor-mediated retention to play a more prominent role. Accumulation of the liposomes in RES organs is expected and is consistent with the behavior of many liposomal and nanoparticle-based systems [43,44]. At the same time, RES accumulation may influence systemic biodistribution and reduce the fraction of administered dose available for tumor targeting. RES sequestration could also affect dosing requirements and treatment schedules, potentially necessitating dose optimization to achieve adequate tumor exposure while minimizing off-target tissue burden. For that reason, potential hepatic and splenic toxicities should be evaluated during preclinical development, particularly with repeated dosing. Potential strategies for future optimization aimed at broadening the therapeutic index by improving circulation time and reducing nonspecific uptake may include modifications to particle size, surface properties, and formulation design with improved tumor-selective delivery. In general, accomplishing deep tumor penetration has become a major research focus aimed at achieving favorable pharmacokinetics without increasing toxicity (reviewed in [56]). Recent trends in overcoming multiple biological barriers for better tumor penetration include personalized and intelligent nanoparticle design that can improve efficacy and patient outcomes (thoroughly reviewed in [57]).

In pharmacokinetics studies, Zn-TL liposomes demonstrated prolonged circulation, low clearance, and a small volume of distribution (Figure 5d), consistent with liposome-bound zinc remaining largely encapsulated during systemic exposure.

Therapeutically, Zn-TL treatment significantly delayed tumor progression during the early and intermediate phases of tumor growth, as evidenced by reduced growth rates (Figure 6b) and prolonged time to reach a five-fold increase in tumor volume (Figure 6c,d). However, this growth-suppressive effect was not sustained at later stages, and no significant differences were observed in time to reach a terminal tumor burden of 2000 mm^3^. These findings suggest suggest that zinc-mediated cytotoxic and metabolic effects are most effective during earlier stages of tumor progression and may be attenuated in advanced tumors characterized by metabolic heterogeneity, hypoxia, altered mitochondrial function, and increased therapeutic resistance [58,59,60].

While the current study provides proof-of-concept support for our approach, several limitations should be acknowledged. First, the relatively low zinc encapsulation efficiency is primarily limited due to the physicochemical properties of zinc sulfate and the passive loading method used for this liposome preparation. During liposome preparation, encapsulation of hydrophilic water-soluble molecules depends primarily on the internal aqueous volume of the liposomes. Zinc sulfate, being a small water-soluble inorganic salt, distributes freely between the internal and external aqueous phases during liposome formation; therefore, only the fraction of total zinc physically trapped within the liposomal core is retained. The small ratio of internal aqueous volume to total preparation volume consequently results in the observed low encapsulation efficiency. Despite this, the formulation delivers a therapeutically relevant intracellular zinc dose, as confirmed by ICP-OES quantification and downstream cytotoxicity, supporting the suitability of this approach for the proposed application. Second, free zinc dosing was reduced by systemic toxicity, limiting direct comparison of high-dose free zinc with Zn-TL. Second, while Zn-TL monotherapy showed clear biological activity, it was insufficient to produce durable tumor control in advanced fast-growing disease.

Collectively, our findings demonstrate that PSMA-targeted liposomal delivery enables selective zinc accumulation in prostate cancer cells, restores zinc-mediated tumor-suppressive mechanisms, and significantly delays tumor growth during early disease stages. The stage-dependent efficacy observed here suggests that Zn-TL may be most effective when used earlier in disease progression. Our results also highlight the potential advantage of using Zn-TL in combination with the standard of care. Targeting carcinogenesis at multiple levels through combination therapy is a fundamental principle of contemporary cancer treatment [61,62], and we intend to incorporate this strategy into future studies. Overall, our future studies will focus on optimizing dosing schedules, evaluating combination strategies, and incorporating early metabolic and apoptotic biomarkers to more fully capture zinc-mediated mechanisms of action.

## Figures and Tables

**Table 1:** Characterization of zinc-loaded liposomes.

Liposomes	Hydrodynamic Diameter (nm)	Polydispersity Index	Zeta Potential (mV)	Zn EE (%)	Zn DLC (%)	Zn DLE (%)
Zn-TL	123.4 ± 0.58	0.20 ± 0.02	−35.59 ± 2.5	3.79 ± 0.27	1.94 ± 0.14	5.66 ± 0.41
Zn-CL	138.3 ± 0.12	0.12 ± 0.00	−34.03 ± 1.53	3.55 ± 0.29	1.83 ± 0.14	5.32 ± 0.42

Abbreviations: TL, PSMA targeting liposome; CL, control liposome; Zn, Zinc; EE, entrapment efficiencies; DLC, drug loading content; DLE, drug loading efficiency.

## Data Availability

The original contributions presented in this study are included in the article/Supplementary Materials. Further inquiries can be directed to the corresponding author.

## Supplementary Materials

The following supporting information can be downloaded at: https://www.mdpi.com/article/10.3390/nano16120705/s1, Figure S1: Characterization of lipid–peptide conjugates; Figure S2: Zn-TL analysis and characterization. Figure S3: Uptake of Dil-labeled Zn-TL liposomes in PC3-PIP cells. Figure S4: Effect of protein corona (PC) formation on PSMA-targeted cellular uptake of Zn-TL liposomes in PC3-PIP cells. Figure S5: Post-therapy biodistribution of Zn-TL.

## Abbreviations

The following abbreviations are used in this manuscript:

Zn	Zinc
ZIP1	Zinc-transporter
PSMA	Prostate-specific membrane antigen
TL	Prostate-specific membrane antigen-targeting liposome
CL	Control liposome
EE	Entrapment efficiency
DLC	Drug loading content
DLE	Drug loading efficiency
PC	Prostate cancer
ATP	Adenosine triphosphate
DSPE	1,2-Distearoyl-sn-glycero-3-phosphoethanolamine
DSPC	1,2-distearoyl-sn-glycero-3-phosphocholine
DOPC	1,2-dioleoyl-sn-glycero-3-phosphocholine
PEG	Polyethylene glycol
DIPEA	N,N-Diisopropylethylamine
NHS	N-Hydroxysuccinimide
PBS	Phosphate-buffered saline
DPBS	Dulbecco’s phosphate-buffered saline
ICP-OES	Inductively coupled plasma optical emission spectrometry
PDI	Polydispersity index
DLS	Dynamic light scattering
PFA	Paraformaldehyde
%RD/g	Percent of recovered dose per gram
RES	Reticuloendothelial system
MRT	Mean residence time
Vd	Volume of distribution
Cl	Clearance
EPR	Enhanced permeability and retention
