# Supplementary material for: Development of PSMA-Targeted Liposomal Zinc for Prostate Cancer Therapy
Source: Nanomaterials (Basel). 2026 Jun 8;16(12):705. doi: 10.3390/nano16120705 (PMC13304933; doi:10.3390/nano16120705)
Supplement: Supplementary file 1 [file nanomaterials-16-00705-s001.zip › nanomaterials-4355934-supplementary.pdf]

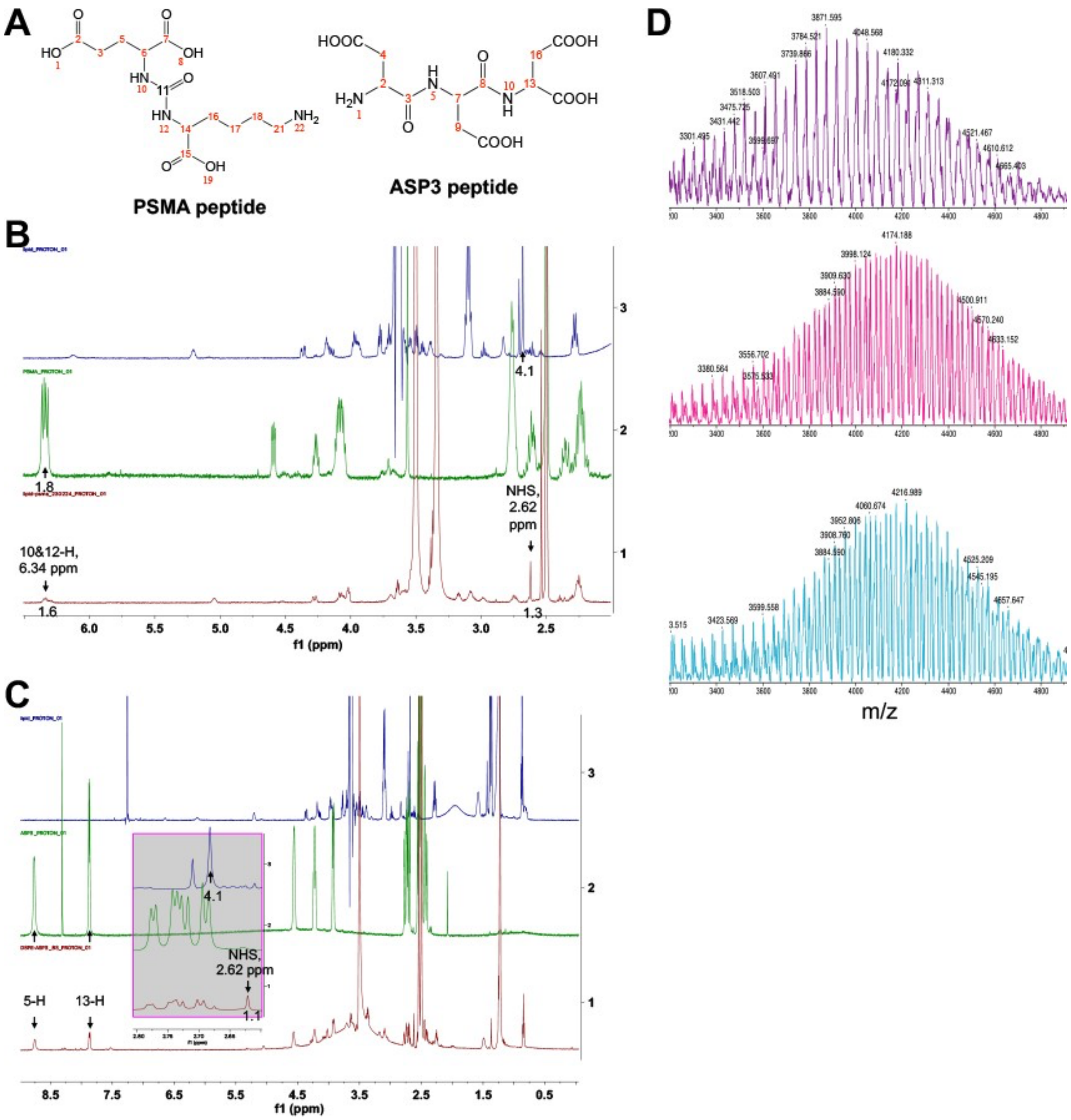

Figure S1: Characterization of lipid-peptide conjugates.
(A) Chemical structure of the PSMA receptor ligand (PSMA peptide) and its control peptide (tri-aspartic acid, ASP3). (B) Overlay of ^{1}H-NMR spectra of DSPE-PEG-NHS lipid (blue, recorded in CDCl$_3$), PSMA peptide (green, recorded in DMSO-d$_6$), and DSPE-PEG-PSMA conjugate (red, recorded in DMSO-d$_6$). Reduction in NHS proton (δ 2.62 ppm) intensity and the appearance of urea proton (10- & 12-H) in the conjugate confirm the formation of DSPE-PEG-PSMA. The proton numbers are mentioned below to their respective spectrum. (C) Overlay of ^{1}H-NMR spectra of DSPE-PEG-NHS (blue, recorded in CDCl$_3$), ASP3 peptide (green, recorded in DMSO-d$_6$) and DSPE-PEG-ASP3 conjugate (red, recorded in DMSO-d$_6$). The reduction in NHS proton intensity, together with the appearance of ASP3 peptide-specific signals (5-H & 13-H) in DSPE-PEG-ASP3 demonstrate the formation of DSPE-EPG-ASP3 conjugate. Inset: magnified region of 2.6-2.8 ppm.(D) The overlay of MALDI-TOF spectrum of DSPE-PEG-NHS (violet), DSPE-PEG-PSMA (pink) and DSPE-PEG-ASP3 (cyan). The average mass of the DSPE-PEG-NHS was ~3.95 kDa, conjugation with peptides resulted in an ~275 Da shift toward higher molecular weight confirming the formation of the conjugates.

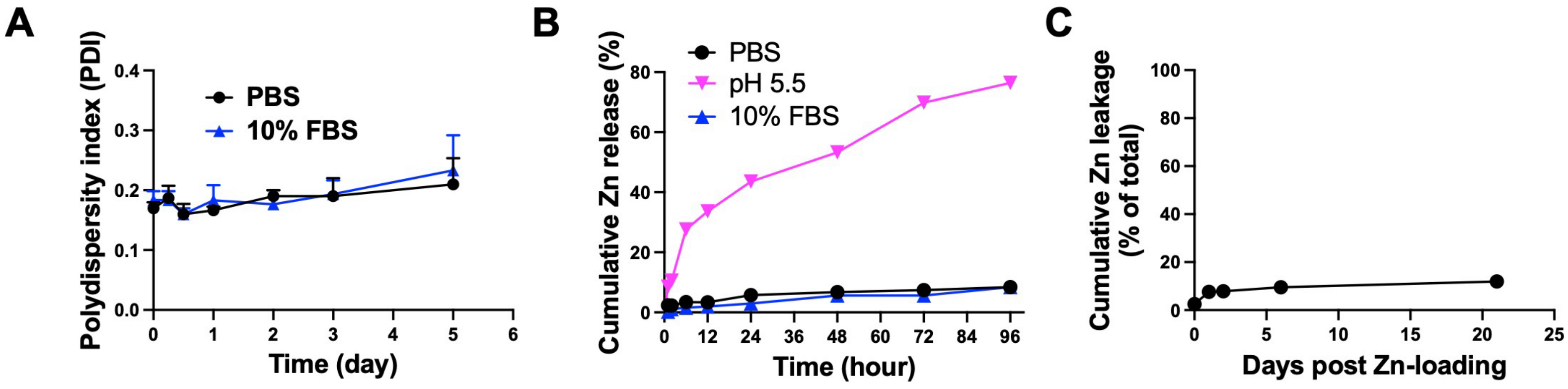

Figure S2: Zn-TL analysis and characterization. (A) Stability evaluation of Zn-TL liposomes by analyzing polydispersity index in PBS and 10% FBS over 5 days. (B) Cumulative zinc release from Zn-TL (DOPC) liposomes tested in three different buffers: PBS, pH 7.4; Acetate buffer, pH 7.4 and 10% FBS in PBS. (C) Percentage of total zinc leaked from liposomes at 4°C over a period of 21 days.

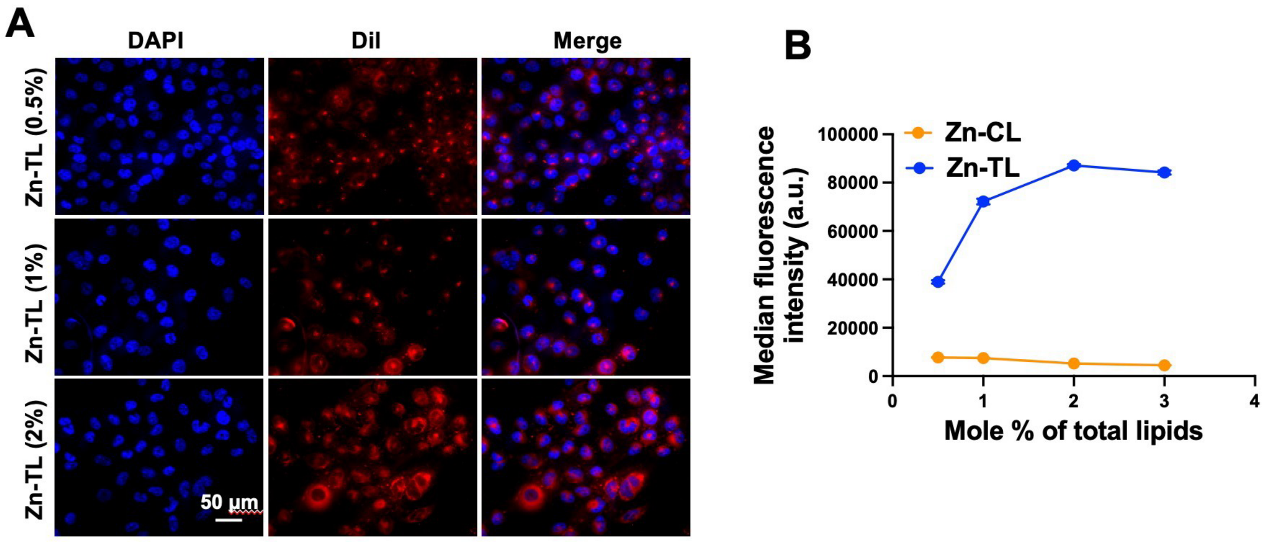

Figure S3: Uptake of Dil-labeled Zn-TL liposomes in PC3-PIP cells. PC3-PIP cells were incubated for 2 hours with Zn-TL liposomes formulated with varying molar percentages of the PSMA-targeting lipid–peptide conjugate (DSPE-PEG-PSMA), while all other lipid components were kept constant. Cellular uptake was assessed qualitatively by fluorescence microscopy (A) and quantitatively by flow cytometry (B). Zn-TL (x%) denotes Dil-labeled Zn-TL liposomes containing x mol% DSPE-PEG-PSMA along with co-lipids.

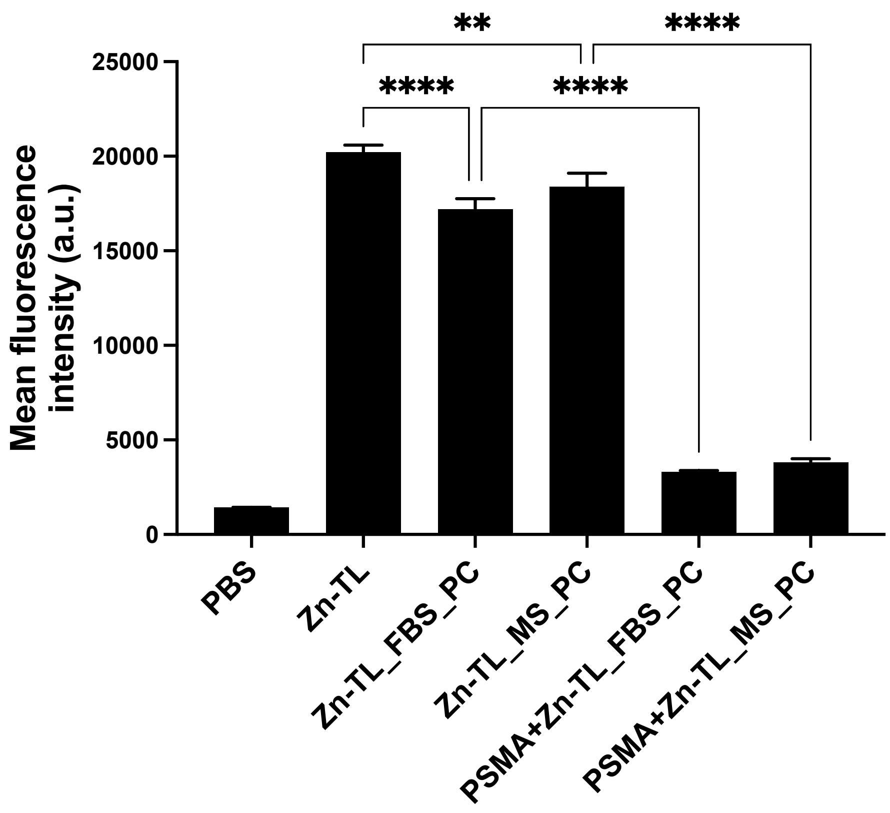

Figure S4. Effect of protein corona (PC) formation on PSMA-targeted cellular uptake of Zn-TL liposomes in PC3-PIP cells. Uptake of Zn-TL liposomes pre-incubated with mouse serum (Zn-TL_MS_PC) or 55% v/v FBS (Zn-TL_FBS_PC). Data are presented as mean ± SD (n = 3). Statistical significance was determined by one-way ANOVA with Tukey's multiple comparison test (**$p < 0.01$, ****$p < 0.0001$).

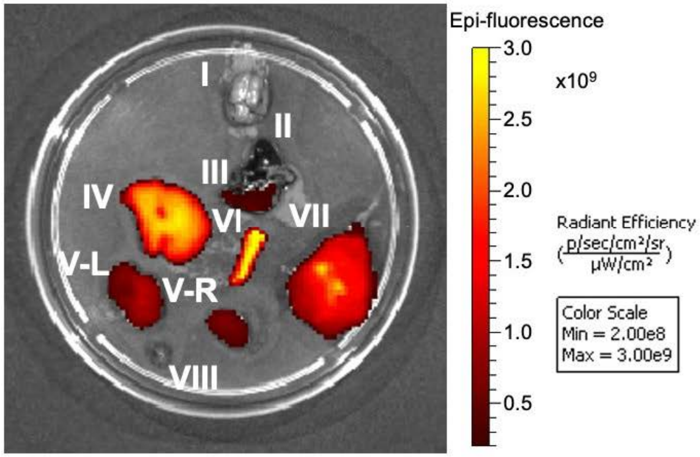

Tissue	Radiant efficiency (p/sec/cm^2/sr)/(µW/cm^2)
Brain (I)	6.93e+08
Heart (II)	3.67e+08
Lung (III)	2.99e+09
Liver (IV)	7.28e+10
Kidney (V)	1.59e+10
Spleen (VI)	1.67e+10
PC3-PIP tumor (VII)	6.13e+10
Muscle (VIII)	2.49e+08

Figure S5: Post-therapy biodistribution of Zn-TL: Representative ex vivo fluorescence imaging (FLI) showing DiR dye signal in a Zn-TL–treated mouse sacrificed upon tumor reaching 2000 mm^3. Corresponding radiant efficiencies for each organ are provided in the Table on the right. I: Brain; II: Heart; III: Lung; IV: Liver; V-L: left kidney; V-R: Right kidney; VI: Spleen; VII: PC3-PIP tumor; VIII: muscle tissue.
